# Comparative Chloroplast Genomics Reveals the Maternal Origin and Evolutionary Relationships of Commercial Pluot Cultivars Within *Prunus*

**DOI:** 10.3390/genes17060607

**Published:** 2026-05-27

**Authors:** Deyin Cao, Xuemei Wen, Zhaoru Guo, Haifang Hu, Bahtiyar Keram, Ming Wang, Yan Wang, Jiaxin Zhang, Zhencan Han, Wenwen Li

**Affiliations:** 1 College of Horticulture, Xinjiang Agricultural University, Urumqi 830052, China; 19893506536@163.com (D.C.);; 2 Xinjiang Xinnongda Environmental Testing Center Co., Ltd., Urumqi 830052, China; 3 College of Computer and Information Engineering, Xinjiang Agricultural University, Urumqi 830052, China; 4 Xinjiang Academy of Forestry Sciences, Urumqi 830063, China; 5 National Tree Seed Base for Economic Forests, Jiamu Experimental Station, Xinjiang Academy of Forestry Sciences, Aksu 843000, China; 6 Engineering Technology Center for Efficient Cultivation and High-Value Utilization of Forest Fruits in Xinjiang, Urumqi 830052, China

**Keywords:** pluots, chloroplast genome, phylogenomics, maternal lineage, molecular markers, divergence time

## Abstract

Background: The phylogenetic placement and chloroplast-inferred maternal relationships of commercial pluot cultivars remain unclear, largely because plastome-level evidence is limited for assessing their affinities with *Prunus salicina* and *Prunus ussuriensis*. Although chloroplast genome structure has been well characterized in angiosperms and in several *Prunus* species, complete plastome resources and comparative genomic evidence for commercial pluot cultivars remain scarce. Methods: Here, we assembled the complete chloroplast genomes of six commercial pluot cultivars and performed comparative genomic, phylogenomic, and divergence time analyses using representative *Prunus* species. Results: All genomes exhibited the typical circular quadripartite structure and ranged from 157,865 to 158,138 bp in length. Genome organization, GC content, and gene content were highly conserved, whereas the IR regions showed an elevated GC content of approximately 42.6%, owing to rRNA gene enrichment. IR boundary comparison revealed contraction at the IRb/SSC boundary in *P. ussuriensis*, while pluot cultivars were structurally more similar to *P. salicina*. In total, 370 SSR loci and four hypervariable regions, namely *rpoB–trnC-GCA*, *petN–psbM*, *trnV-UAC–trnM-CAU*, and *trnP-UGG–psaJ*, were identified as candidate molecular markers for *Prunus* germplasm identification and genetic analysis. Phylogenomic analysis resolved four major clades within *Prunus* and showed that ‘Flavor King’, ‘Flavor Supreme’, and ‘Flavor Queen’ grouped with *P. ussuriensis*, whereas ‘Flavorosa’, ‘Dinosaur Egg’, and ‘Flavorich’ grouped with *P. salicina*. Conclusion: Overall, this study provides the first comparative plastome analysis of six commercial pluot cultivars and offers chloroplast-level evidence for their maternal affinities within *Prunus*, together with useful marker resources for cultivar identification and germplasm evaluation.

## 1. Introduction

The commercial pluot cultivars hereafter referred to as pluots belong to the genus *Prunus* within the Rosaceae family and are perennial tree fruit crops with significant economic value. These cultivars are characterized by distinctive fruit flavor and high tolerance to cold and drought stress. They show excellent ecological adaptability in regions such as North China, Northwest China, and the Tarim Basin area of Xinjiang, where they have become key fruit tree germplasm resources for improving fruit quality and production efficiency in arid and semi-arid areas [1,2]. In modern fruit cultivation, many superior cultivars known as “pluots” are commonly regarded as artificially bred interspecific hybrids between plums and apricots [3]. However, their breeding histories often include complex multi-parent hybridization and backcrossing to introduce specific agronomic traits (e.g., cold tolerance and distinctive flavor), resulting in highly intricate genetic backgrounds [4]. Given the complex reticulate evolution, frequent interspecific hybridization, and common polyploidy within *Prunus*, relying solely on morphological characteristics is insufficient to clarify the precise maternal genetic background of these commercial cultivars. Chloroplast genomes are typically maternally inherited in angiosperms and generally exhibit limited recombination, making them valuable tools for tracing the maternal ancestry of complex hybrids. Therefore, characterizing the chloroplast genomes of pluots can help clarify their taxonomic position and provide insights into the history of germplasm introgression underlying modern cultivated varieties, thereby providing an important basis for accurate germplasm conservation and genetic improvement.

The chloroplast genome (cpDNA) exhibits predominantly uniparental inheritance, structural conservation, and moderate evolutionary rates. It largely reduces interference from recombination and nuclear paralogous sequences, making it a valuable source of molecular markers for resolving plant phylogenetic relationships, inferring species divergence times, and investigating adaptive evolution [5,6]. In recent years, with the widespread adoption of high-throughput sequencing technologies, comparative chloroplast genomics has emerged as a powerful tool for resolving complex taxonomic issues within the genus *Prunus*. For instance, Feng et al. used cpDNA evidence to demonstrate significant positive selection on multiple genes, such as *atpE* and *ccsA*, within *Prunus*, and clarified the phylogenetic relationship between *Prunus cistena* and *Prunus jamasakura* [7]. Duan et al. clarified the taxonomic positions of *Prunus mongolica* and *Prunus pedunculata* based on cpDNA phylogenetic trees [8]. The complete chloroplast genome sequencing of *Prunus zhengheensis* revealed a conserved genomic structure compared with seven other *Prunus* species, while also identifying six species-specific genes, including *rps18* and *matK*. Phylogenetic and morphological evidence collectively indicated that it forms a recently diverged sister group to *Prunus mume*, providing crucial data for studies of *Prunus* systematics and evolution [9]. Cui et al. reported the first chloroplast genomes of three wild *Prunus* species, namely *Prunus armeniaca*, *Prunus divaricata*, and *Prunus tianshanica*. They found that sequence variation was primarily concentrated in non-coding regions and identified seven highly variable regions, including *petN-psbM* and *ndhC-trnV*. Additionally, their results indicated that *P. armeniaca* and *Prunus mandshurica* are more closely related to each other than to *P. mume*, while *P. divaricata* and *P. tianshanica* belong to the subgenera *Prunus* and subgenus *Microcerasus*, respectively [10]. These studies demonstrate that chloroplast genomic data are particularly useful for clarifying ambiguous species boundaries among closely related species within *Prunus* and for elucidating potential hybridization histories.

Although chloroplast genomes of several *Prunus* species have been reported, systematic studies on the complete chloroplast genomes of commercial pluot cultivars and their evolutionary relationships with closely related species such as *P. salicina* and *P. ussuriensis* remain scarce. In particular, plastome-level evidence for tracing the maternal background of commercial pluot cultivars is still limited, despite their complex breeding history involving interspecific hybridization and backcrossing. Therefore, this study aimed to assemble and compare the complete chloroplast genomes of six commercial pluot cultivars and to evaluate their maternal affinities with representative *Prunus* species. To our knowledge, this study provides the first comparative analysis of complete chloroplast genomes from six commercial pluot cultivars. These data offer new plastome resources for this complex hybrid group, help clarify their maternal affinity, and provide candidate chloroplast markers for germplasm identification and future genetic studies.

## 2. Materials and Methods

### 2.1. Plant Materials, DNA Extraction, and Sequencing

This study analyzed a total of 28 chloroplast genomes, including 22 representative species of *Prunus* L. for which chloroplast genome sequences were downloaded from the NCBI database [11] (Table S1). Additionally, chloroplast genome sequencing and assembly were performed for six commercial pluot cultivars (Figure S1), namely: ‘Flavorosa’, ‘Flavor Queen’, ‘Flavor King’, ‘Flavorich’, ‘Dinosaur Egg’, and ‘Flavor Supreme’. All tested materials were commercially cultivated varieties derived from interspecific hybrids between plum and apricot. These cultivars were selected because they are representative commercial pluot cultivars that are widely cultivated and/or marketed and exhibit distinct differences in fruit appearance, ripening period, and horticultural characteristics. Fresh young leaves were collected from the Jiamu Experimental Station in Wensu County, Aksu, Xinjiang China, affiliated with the Xinjiang Academy of Forestry Sciences. Samples were immediately frozen in liquid nitrogen upon collection and subsequently stored at −80 °C for subsequent total DNA extraction, library construction, and sequencing.

Total DNA was extracted using a plant genomic DNA kit according to the manufacturer’s instructions (Tiangeng Biotech Co., Ltd., Beijing, China). DNA concentration and purity were assessed using a NanoDrop 2000 micro-volume spectrophotometer (Thermo Fisher Scientific, Wilmington, DE, USA) and 1% agarose gel electrophoresis. Qualified DNA was fragmented, end-repaired, and used for library construction (Figure S2). Library construction and subsequent sequencing on the Illumina HiSeq platform (Illumina, Inc., San Diego, CA, USA) were performed by Wuhan Benna Technology Co., Ltd. (Wuhan, China).

### 2.2. Genome Assembly and Annotation of cpDNA

Using the clean sequencing data, chloroplast genome assembly was performed with GetOrganelle v.1.7.5 software (GetOrganelle development team, Kunming, China) [12] using the following command: get_organelle_from_reads.py -1 forward.fq -2 reverse.fq -o plastome_output -R 15 -k 21,45,65,85,105 -F embplant_pt. Following preliminary assembly, manual correction and sequence joining were performed using the Geneious Prime v.2025.2 software (Biomatters Ltd., Auckland, New Zealand; http://www.geneious.com/; accessed on 17 March 2025) with the pluot cultivar ‘Weiwang’ (GenBank accession No. MW406463.1) as the reference sequence, thereby generating complete chloroplast genome sequences [13]. Genome annotation was performed using the GeSeq v.2.03 online tool (Max Planck Institute of Molecular Plant Physiology, Potsdam-Golm, Germany; accessed on 17 March 2025; https://chlorobox.mpimp-golm.mpg.de/geseq.html) [14], and circular chloroplast genome maps were generated using Chloroplot v.0.2.4 (https://irscope.shinyapps.io/Chloroplot/; accessed on 21 July 2025) [15].

### 2.3. Comparative Analysis of Cp Genome in Five Prunus Species

#### 2.3.1. Selection of Comparative Genomes

To investigate plastome variation and evolutionary differences among *Prunus* taxa, this study selected five representative *Prunus* chloroplast genomes for comparative analysis. Since the six pluot cultivars sequenced and assembled in this study belong to the same hybrid group and showed highly conserved chloroplast genomic sequences, the pluot cultivar ‘Flavor Supreme’ was selected as a representative pluot plastome. It was then compared with four other *Prunus* species obtained from the NCBI database, namely *P. salicina*, *at*, *Prunus sibirica*, and *P. armeniaca*.

#### 2.3.2. Method for Codon Usage Analysis

Codon usage bias refers to the unequal use of synonymous codons among genes or genomes and may vary among species or lineages. This characteristic is commonly quantified using relative synonymous codon usage (RSCU) [16]. To evaluate codon usage patterns across the selected plastomes, an online bioinformatics platform (Genepioneer Cloud, online version; Nanjing Genepioneer Biotech Co., Ltd., Nanjing, China; accessed on 21 July 2025; http://112.86.217.82:9929/#/tool/alltool/detail/287) was utilized to calculate RSCU values. RSCU values were interpreted as follows: RSCU = 1 indicates no codon usage bias; RSCU > 1 indicates that a codon is used more frequently than expected; and RSCU < 1 indicates that a codon is used less frequently than expected.

#### 2.3.3. Method for IR Boundary Analysis

The IR region contributes to chloroplast genome stability through homologous recombination, and variation in IR/SC junctions is considered one of the major factors underlying chloroplast genome size differences [17]. This study utilized IRscope v.0.1 (https://irscope.shinyapps.io/irapp/; accessed on 28 July 2025) and R scripts implemented in R v.0.1.R (R Foundation for Statistical Computing, Vienna, Austria) [18] to visualize and compare the expansion and contraction of the IR regions in the cp genomes of five *Prunus* taxa.

#### 2.3.4. Repeat Sequence Analysis

Simple sequence repeats (SSRs) were detected using the MISA online tool v.2.1 (Leibniz Institute of Plant Genetics and Crop Plant Research, Gatersleben, Germany; https://webblast.ipk-gatersleben.de/misa/; accessed on 28 August 2025) [19]. The minimum numbers of repeat units for mono-, di-, tri-, tetra-, penta-, and hexanucleotide SSRs were set to 10, 5, 4, 3, 3, and 3, respectively; and the maximum interval between two SSRs was set to 50 bp [20]. Long repeat sequences, including forward, reverse, complement, and palindromic repeats, were analyzed using REPuter v.2 (Bielefeld University, Bielefeld, Germany; https://bibiserv.cebitec.uni-bielefeld.de/reputer; accessed on 30 August 2025) [21]. Parameters were set as follows: minimum repeat length (minimum) 30 bp, Hamming distance of 3, and maximum repeat length of 5000 bp [22].

#### 2.3.5. Full-Sequence Alignment and Nucleotide Diversity

The cp genomes of five *Prunus* taxa were subjected to whole-genome alignment using mVISTA v. 2.0 software (Lawrence Berkeley National Laboratory, Berkeley, CA, USA; https://genome.lbl.gov/vista/mvista/submit.shtml; accessed on 2 September 2025) in Shuffle-LAGAN mode [23]. Multiple sequence alignment was performed with MAFFT v.7.505 (Research Institute for Microbial Diseases, Osaka University, Osaka, Japan) [24], and nucleotide polymorphism (Pi) was calculated using DnaSP v.6.12.03 (University of Barcelona, Barcelona, Spain) [25]. A sliding window size of 600 bp and a step size of 200 bp were set [20] to identify highly variable and conserved regions within the genomes.

#### 2.3.6. Phylogenetic Analysis

To resolve the phylogenetic relationships among *Prunus* taxa and their closely related groups, phylogenetic trees were constructed using Bayesian inference (BI) and maximum likelihood (ML) methods based on complete chloroplast genome sequences. Sequences were aligned using MAFFT v.7.505 software [24], with *Malus prattii* and *Crataegus pinnatifida* used as outgroups. Because chloroplast genomes are generally inherited as a single linked unit with limited recombination, the complete chloroplast genome alignment was treated as a single integrated plastome dataset to infer chloroplast-inferred maternal affinities among the sampled taxa. The ML tree was constructed using IQ-TREE v.2.2.5 software (IQ-TREE Development Team, Vienna, Austria) [26], with the best-fit model TVM + F + R2 selected by ModelFinder [27] and branch support assessed using 1000 bootstrap replicates. The BI tree was constructed using MrBayes v.3.1.2 (MrBayes Development Team, Stockholm, Sweden) [28] under the GTR + G model. The MCMC analysis was run for 1,000,000 generations, with sampling every 100 generations. Convergence was determined when the standard deviation of split frequencies was less than 0.01. The first 25% of samples were discarded as burn-in, and a majority-rule consensus tree was generated from the remaining samples.

#### 2.3.7. Estimation of Divergence Time

Divergence times were estimated using the MCMCTREE program implemented in PAML v.4.9 (University College London, London, UK) [29]. The topology of the maximum likelihood tree was used as the input tree, and the GTR model was applied for molecular dating analysis. Four secondary calibration constraints were obtained from the TimeTree database v.5.0 (Temple University, Philadelphia, PA, USA; http://www.timetree.org/) [30]: the divergence between *Prunus padus* and *C. pinnatifida* was constrained to 34.4–67.2 Mya; the divergence between *P. armeniaca* and *Prunus avium* was constrained to 7.4–58.2 Mya; the divergence between *P. mume* and *Prunus humilis* was constrained to 33.7–61.5 Mya; and the divergence between *P. ussuriensis* and *P. salicina* was constrained to 33.7–61.5 Mya.

The MCMC chain length was set to 1,010,000 generations, with sampling every 10 generations. The first 10,000 generations were discarded as burn-in, resulting in 100,000 retained samples. The convergence of the MCMC analysis was assessed by checking the effective sample size and the stability of posterior estimates. The results were visualized using FigTree v.1.4.2 (University of Edinburgh, Edinburgh, UK) [31,32].

## 3. Results

### 3.1. Assembly and Annotation of the cp Genome

**Figure 1:**
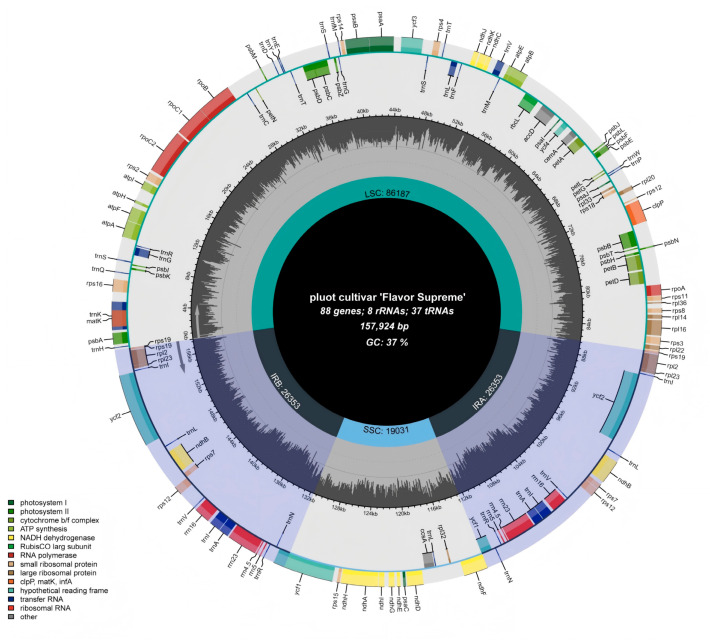
Chloroplast genome atlas of pluot cultivar ‘Flavor Supreme’. Species names and specific information about the genome (length, GC content and number of genes) are shown in the center of the figure. Genes with different functions are indicated by different colors. Dark gray inside corresponds to GC content, light gray to AT content.

Using Illumina sequencing technology, this study successfully generated clean sequencing data for the chloroplast genomes of six commercial pluot cultivars: ‘Flavor Queen’, ‘Flavorosa’, ‘Dinosaur Egg’, ‘Flavor Supreme’, ‘Flavor King’, and ‘Flavorich’. The assembly results showed that the chloroplast genomes of these six cultivars ranged from 157,916 to 157,924 bp in size, and all exhibited a typical circular quadripartite structure (Figure 1, Figure S1). Among them, ‘Flavor Queen’, ‘Flavor King’, and ‘Flavor Supreme’ shared identical sequence lengths of 157,924 bp, while ‘Flavorosa’, ‘Dinosaur Egg’, and ‘Flavorich’ had the same genome length of 157,916 bp. Each genome consisted of one large single-copy (LSC, 86,123–86,187 bp), one small single-copy (SSC, 19,029–19,031 bp), and two inverted repeat regions (IRs, 26,353–26,382 bp) (Table S2).

In terms of GC content, ‘Flavor Queen’, ‘Flavor King’, and ‘Flavor Supreme’ had an overall GC content of 36.72%, while ‘Flavorosa’, ‘Dinosaur Egg’, and ‘Flavorich’ each showed all overall GC content of 36.75%. The overall GC content was lower than the AT content, consistent with the general characteristic of angiosperm chloroplast genomes being AT-rich. Furthermore, the GC content in the IR region (42.59–42.62%) was significantly higher than that in the LSC region (34.51–34.58%) and SSC region (30.37–30.39%), indicating that the IR regions were more conserved than the single-copy regions [17].

Comparative annotation analysis of the chloroplast genomes of five *Prunus* taxa (Table 1) revealed that the total number of genes ranged from 131 to 132. The number of protein-coding genes ranged from 85 to 86, with this variation primarily attributed to copy number differences in the *rps19* gene. The number of tRNA genes ranged from 37 to 39, mainly due to the presence or absence of the *trnS-GGA* gene and copy number variations in the *trnS-GCU* gene. The number of rRNA genes was highly conserved, with eight rRNA genes detected in each chloroplast genome.

Gene deletion and pseudogenization events were also observed: *infA* and *trnR* were detected only in *P. ussuriensis*; *rps19* was identified as a pseudogene in *P. salicina*; *ycf1* was a pseudogene in *P. sibirica* and *P. armeniaca*. Functional annotation categorized genes into four groups: self-replication, photosynthesis, other functions, and unknown functions. Structural analysis revealed 19 duplicated genes located in the inverted repeat (IR) regions. Intron analysis revealed 15 genes containing one intron (*ndhA*, *ndhB*, *petB*, *petD*, *atpF*, *rpl16*, *rpl2*, *rps16*, *rpoC1*, *trnA-UGC*, *trnG-GCC*, *trnI-GAU*, *trnK-UUU*, *trnL-UAA*, *trnV-UAC*), while three genes contained two introns (*rps12*, *clpP*, *ycf3*) (Table 2).

### 3.2. Analysis of Codon Preferences

**Figure 2:**
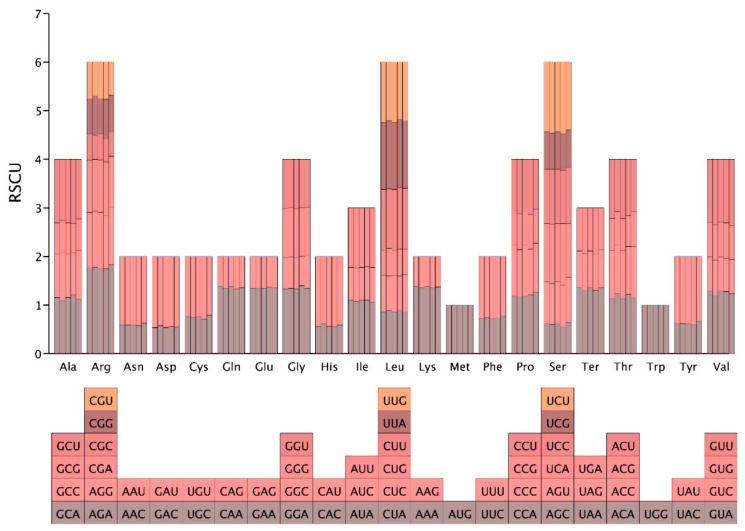
Histograms were used for the relative synonymous codons of the five *Prunus* spp. The upper columns describe the total RSCU values for the 20 amino acids, and each block below represents a different codon encoding an amino acid; the five columns are arranged in the following order: pluot cultivar ‘Flavor Supreme’, *P. sibirica*, *P. ussuriensis*, *P. armeniaca*, *P. salicina*.

Codon usage bias is influenced by multiple factors including genomic base composition, translational selection pressures, and functional requirements, making it a crucial feature associated with gene expression regulation and evolutionary adaptation [13]. In this study, the analysis showed that the chloroplast genomes of *P. sibirica*, *P. ussuriensis*, *P. armeniaca*, *P. salicina*, and the pluot cultivar ‘Flavor Supreme’ encode 52,740, 52,639, 52,650, 52,638, and 52,641 codons, respectively. These five *Prunus* taxa collectively contain 61 sense codons encoding 20 amino acids (Figure 2).

Among these, leucine (Leu) exhibited the highest abundance, with 5243 to 5570 codons, accounting for 9.96% to 10.58% of the total. Serine (Ser) and arginine (Arg), both encoded by six codons, were represented by 4418–4711 codons (8.39–8.95%) and 3159–3315 codons (6.0–6.3%), respectively. Tryptophan (Trp) and methionine (Met) exhibited the lowest usage frequencies, with only 669–745 instances (1.27–1.42%) and 853–860 instances (1.62–1.63%), respectively (Table S3).

**Figure 3:**
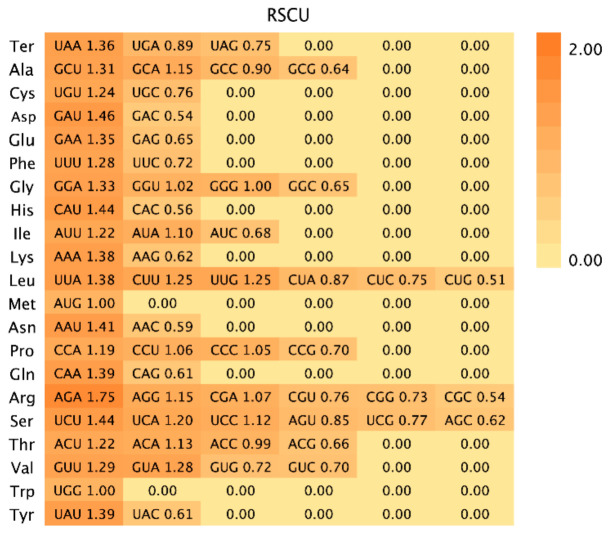
The codon usage rates of the cp genomes of five *Prunus* spp. The colors in the figure indicate ranges of RSCU values, and the codon usage rates increase as the color changes from light yellow to orange.

At the individual codon level, AGA exhibited the highest usage frequency, while CUG showed the lowest (Figure 3). RSCU analysis revealed 32 high-frequency codons (RSCU > 1), of which 24 ended with A/U (75%); 29 low-frequency codons (RSCU < 1) were identified, of which 25 ended with G/C (86.2%). These results indicate a significant preference for A/U-terminating codons in the chloroplast genomes of these five *Prunus* species.

### 3.3. IR Boundary Analysis

**Figure 4:**
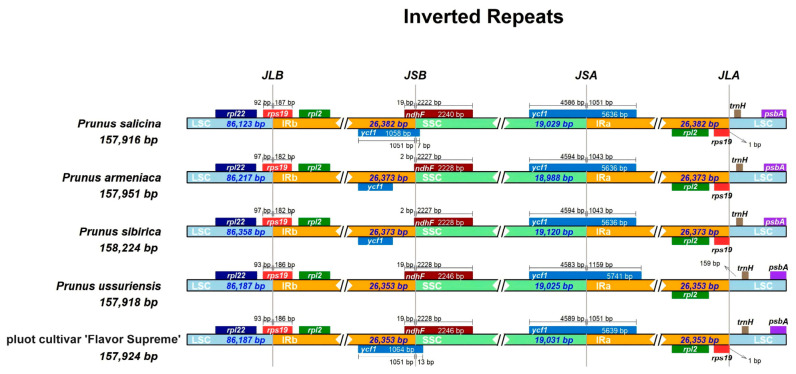
Comparison of IR boundaries in the cp genomes of five *Prunus* species. LSC, SSC, and IR region are shown. The distance between each boundary endpoint and adjacent genes is indicated above the central line in base pairs (bp).

The cp genome sizes of the five *Prunus* taxa were similar (157,916–158,224 bp), and the gene distribution patterns in the IR boundary regions were largely conserved (Figure 4). Genes such as *rpl22*, *rps19*, *rpl2*, *ndhF*, *ycf1*, *psbA*, and *trnH* were located at the boundaries of all species, indicating a high level of conservation in the IR boundary regions of *Prunus* cp genomes.

Despite high conservation in gene types, subtle variations in gene length at the boundaries may reflect evolutionary differentiation among species. Specifically, the IR boundary characteristics of pluot cultivars and *P. salicina* were highly similar, whereas those of *P. armeniaca* and *P. sibirica* also showed remarkable similarity. The primary differences between these two groups occurred at the IRb/SSC boundary, as indicated by varying degrees of expansion in the *ycf1* and *ndhF* genes. Notably, *P. ussuriensis* differed markedly from the other four taxa: it lacked partial copies of the *ycf1* copy at the IRb/SSC boundary and the *rps19* copy at the IRa/LSC boundary. Additionally, the *ycf1* gene at its SSC/IRa boundary was approximately 100 bp longer than in the other four taxa, suggesting that this species may have experienced unique structural variations in its chloroplast genome.

### 3.4. Analysis of Repetitive Sequences and SSRs

**Figure 5:**
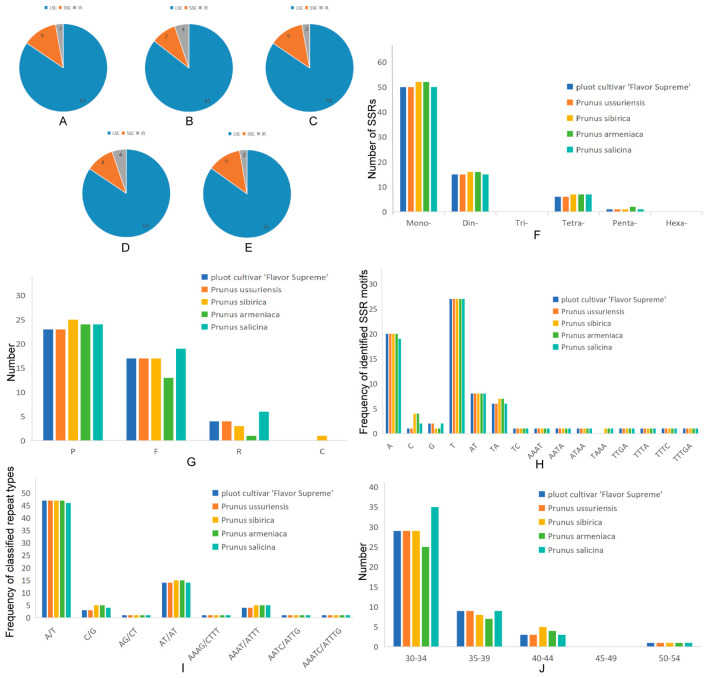
Analysis of repetitive sequences in the chloroplast genomes of five *Prunus* species. (**A**) Number of SSRs distributed in different copy regions of the pluot cultivar ‘Flavor Supreme’; (**B**) number of SSRs distributed in different copy regions of *P. sibirica*; (**C**) number of SSRs distributed in different copy regions of *P. ussuriensis*; (**D**) number of SSRs distributed in different copy regions of *P. armeniaca*; (**E**) number of SSRs distributed in different copy regions of *P. salicina*; (**F**) number of the six SSR types; (**G**) types of long sequence repeats; (**H**) number of different SSR repeat unit types; (**I**) frequency of repeat types classified by sequence complementarity; (**J**) frequency of each type calculated by length.

A total of 370 SSR loci were identified across the cp genomes of the five *Prunus* taxa. Among these, mononucleotide repeats were the most abundant type (254 loci, accounting for 68.65%), followed by dinucleotide repeats (77 loci, 20.81%) and tetranucleotide repeats (33 loci, 8.92%); pentanucleotide repeats were the least abundant (6 loci, 1.62%). Trinucleotide and hexanucleotide repeats were not detected (Figure 5F).

The distribution patterns of SSRs across the four regions—LSC, SSC, IRa, and IRb—were generally consistent among the five taxa: SSRs were predominantly enriched in the LSC region (84.42–85.53%), followed by the SSC region (9.21–12.68%), while the IRa and IRb regions contained relatively fewer SSRs and showed similar distribution patterns (2.82–5.26%) (Figure 5A–E). Overall, SSR sequences predominantly featured A/T nucleotides, with over half of the SSRs consisting of mononucleotide repeats composed of A or T, reflecting the strong AT bias of chloroplast genomes.

Comparisons among taxa revealed that the highest proportion of mononucleotide repeats was observed in the pluot cultivar ‘Flavor Supreme’ and *P. ussuriensis* (69.44% in both), followed by the *P. salicina* (68.49%), *P. sibirica* (68.42%), and *P. armeniaca* (67.53%). Among the mononucleotide repeats, the T type accounted for the highest proportion; the A type occurred 20 times in four taxa, whereas *P. salicina* contained 19 occurrences. Additionally, TAAA repeats were not detected in the pluot cultivar ‘Flavor Supreme’ or *P. ussuriensis* (Figure 5H). Among dinucleotide and longer repeat types, commen repeat motifs, such as A/T, C/G, and AG/CT were shared by all five cp genomes, indicating limited interspecific variation (Figure 5I).

Analysis of long repeated sequences identified a total of 221 repeats, including 119 palindromic repeats, 83 forward repeats, 18 reverse repeats, and 1 complementary repeat (Figure 5G). Among these, palindromic repeats were most abundant in *P. sibirica* (25 repeats), followed by *P. armeniaca* and *P. salicina* (24 repeats each), and ‘Flavor Supreme’ and *P. ussuriensis* (23 repeats each). Forward repeats were most abundant in *P. salicina* (19 repeats), while *P. armeniaca* had the fewest (13 repeats). Complementary repeats were detected only in *P. sibirica* (1 repeat) and were absent in the other four taxa. Repeat lengths predominantly clustered within the range of 30–34 bp (147 repeats, 66.52%), with no repeats detected in the 45–49 bp range (Figure 5J).

### 3.5. Comparative Analysis of Cp Genome Sequences and Nucleotide Diversity

**Figure 6:**
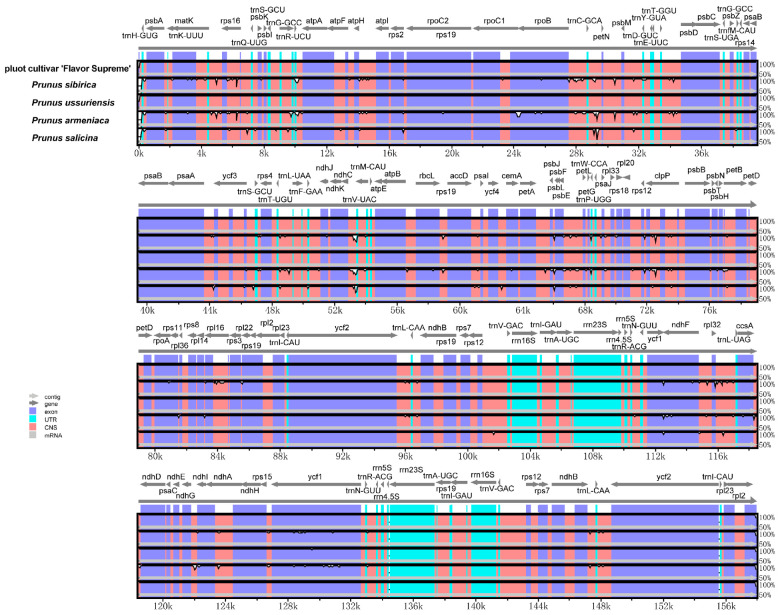
Sequence identity plots of the cp genome sequences of five *Prunus* species. Gray arrows indicate gene orientation, red bars indicate non-coding sequences, purple bars indicate exons, and blue bars indicate introns; vertical scales indicate percent identity in the range of 50~100%.

Complete cp genome sequence alignments enable the accurate identification of sequence variation patterns among species. The mVISTA visualization of the alignment results showed that the cp genomes of the five *Prunus* taxa were highly conserved, with sequence variation predominantly concentrated in non-coding regions (Figure 6).

**Figure 7:**
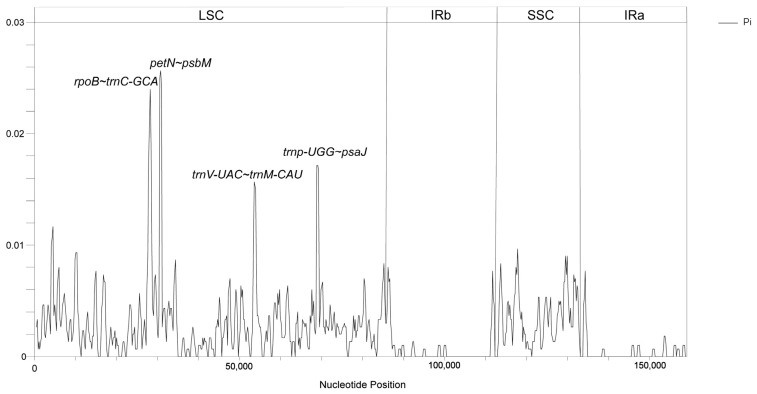
Sliding window analysis pi in the cp genomes of five *Prunus* species. The x-axis represents the position of the midpoint of each window, and the y-axis represents the nucleotide diversity in each window.

Nucleotide diversity (Pi), calculated using DnaSP v.6, ranged from 0 and 0.028. The levels of variation in the LSC region were significantly higher than those in the IR and SSC regions. Four highly variable regions (Pi > 0.015) were identified within the LSC region: *rpoB~trnC-GCA*, *petN~psbM*, *trnV-UAC~trnM-CAU*, and *trnP-UGG~psaJ* (Figure 7). These highly variable regions may serve as potential molecular markers, providing valuable references for species identification and phylogenetic studies in *Prunus* species.

### 3.6. Phylogenetic Tree Construction

**Figure 8:**
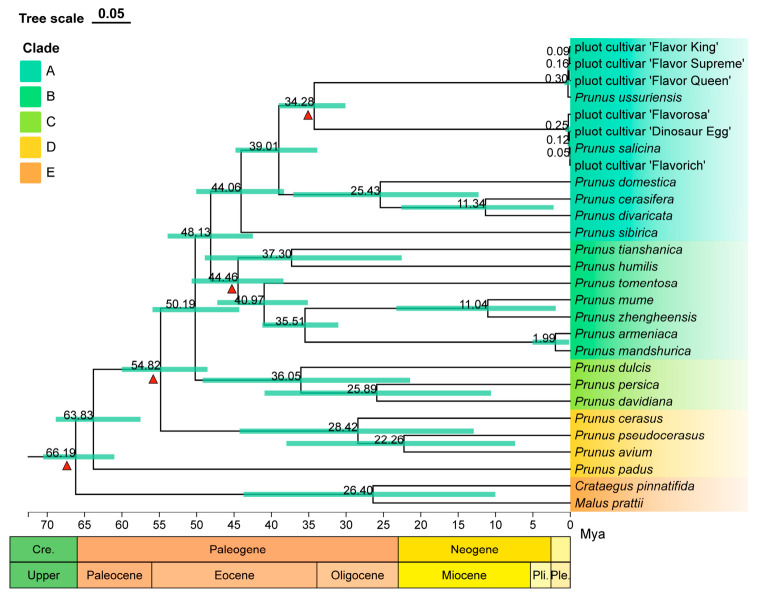
Estimation of differentiation time based on the ML tree of *Prunus* species. The green line indicates the 95% highest posterior density and the red triangles indicate three calibration points.

To investigate the phylogenetic position of pluot cultivars, we selected *C. pinnatifida* and *M. prattii* as outgroups and constructed a phylogenetic tree based on the chloroplast genomes of 28 Rosaceae species. The phylogenetic trees inferred using Bayesian inference (BI) and maximum likelihood (ML) methods showed identical topologies (Figure 8, Figure S3) with exceptionally high node support, indicating the high robustness of the phylogenetic inference.

Phylogenetic analysis resolved all species into five major clades. Among them, the ‘Flavor Supreme’, ‘Flavor King’, and ‘Flavor Queen’ clustered with *P. ussuriensis*; while ‘Flavorosa’, ‘Dinosaur Egg’, and ‘Flavorich’ clustered with the *P. salicina*. *P. armeniaca* and *P. sibirica* formed another clade, indicating their relatively distant relationship with the pluot cultivars.

The correspondence between phylogenetic clades and subgenus classification, together with the estimated divergence times, is as follows: Clade A corresponds to subgenus *Prunus*, with a crown group age of 44.06 Mya (95% HPD: 38.23–50.06 Mya); Clade B corresponds to subgenus *Armeniaca*, with a crown group age of 44.46 Mya (95% HPD: 38.40–50.67 Mya); Clade C corresponds to subgenus *Amygdalus*, with a crown group age of 36.05 Mya (95% HPD: 21.44–49.20 Mya); Clade D corresponds to subgenus *Cerasus*, with a crown group age of 28.42 Mya (95% HPD: 12.92–44.25 Mya); and Clade E represents the outgroup. These time estimates are based on chloroplast genomic data and primarily reflect the evolutionary history of the maternal lineage.

## 4. Discussion

### 4.1. Genome Structure and Sequence Conservation

This study assembled the chloroplast genomes of six pluot cultivars and conducted a comparative genomic analysis with four other representative *Prunus* species. The results showed that the chloroplast genomes of the examined taxa exhibited the typical angiosperm quadripartite structure, with highly consistent genome lengths (157,865–158,131 bp), gene counts (131–132 genes), and total GC content. These characteristics are largely consistent with the previously reported cp genome of the pluot cultivar ‘Flavor King’ [33]. Notably, the GC content in the IR region (42.57–42.62%) was significantly higher than those in the LSC region (34.51–34.58%) and SSC region (30.30–30.50%). This high GC content can primarily be attributed to the enrichment of rRNA genes (*rrn16*, *rrn23*, *rrn4.5*, *rrn5*) with high GC content in this region [34]. The highly similar genomic structures among the examined taxa further confirm the evolutionary conservation of the cp genome in *Prunus* species [35].

In addition to GC-content effects, the structural stability and low sequence divergence of the IR regions may also be explained by plastid DNA repair mechanisms. Recent studies have emphasized that homologous recombination is a major pathway for repairing double-strand breaks in plastid genomes, and the two highly similar IR copies can serve as reciprocal homologous templates for accurate repair. This repair-mediated homogenization may contribute to the strong conservation and reduced substitution rates commonly observed in IR regions compared with the LSC and SSC regions. Moreover, the high copy number of plastid genomes within plant cells provides abundant intact templates for homologous recombination, thereby increasing repair efficiency and reducing the probability of mutation accumulation. These mechanisms may help explain why the pluot plastomes examined here are highly conserved at both structural and sequence levels. Compared with plant mitochondrial genomes, which often show more extensive recombination-associated rearrangements and structural dynamism, plastid genomes—especially their IR regions—appear to be subject to stronger repair-mediated stabilization [36,37].

### 4.2. Codon Preference and IR Boundary Variation

Codon preference reflects adaptive selection for translation efficiency and accuracy during the long-term evolution of species [38]. In this study, the cp genomes of five *Prunus* taxa encoded 20 amino acids using 61 codons, with leucine being the most frequent and cysteine being the least frequent. RSCU analysis revealed that high-frequency codons (RSCU > 1) predominantly ended with A/U, whereas low-frequency codons were more likely to the end with G/C ending codons. This A/U preference pattern is consistent with findings in *Malus* [39], *Caragana* [40], and most dicotyledons [41], indicating that AT richness in the cp genome strongly shapes codon usage.

Boundary variations, including expansion and contraction of the IR regions, are primary drivers of cp genome size differences among angiosperms [42]. This study revealed significant IR boundary variations in *P. ussuriensis* compared with *P. salicina* and the pluot cultivar ‘Flavor Supreme’: the IRb/SSC boundary lacked a portion of the *ycf1* copy, while the IRa/LSC boundary lacked the *rps19* copy. In contrast, the ycf1 gene in *P. armeniaca* and *P. sibirica* was entirely located within the SSC region, without any boundary extension. Although the cp genome structure is generally conserved across most land plants, minor boundary shifts between genera or even among closely related species, such as the positional changes in *rps19* and *ycf1*—often provide phylogenetically significant information [43,44].

### 4.3. Screening of Repeat Sequences and Highly Variable Regions

Simple sequence repeats (SSRs), as highly polymorphic molecular markers characterized by high mutation rates and genetic stability, are widely used in population genetics and phylogenetic studies [45,46]. This study identified 370 SSRs across five *Prunus* cp genomes, most of which consisted of A/T-rich motifs, with no trinucleotide or hexanucleotide repeats detected. The high similarity in SSR quantity and distribution patterns between the pluot cultivar ‘Flavor Supreme’ and *P. ussuriensis* suggests an extremely close phylogenetic relationship between them. Furthermore, mVISTA and nucleotide diversity (Pi) analyses indicated that sequence variation was primarily concentrated in the LSC and SSC regions, while the IR region exhibited the highest conservation. This pattern is consistent with the proposed role of IR sequences in homologous recombination-mediated plastid DNA repair, which may constrain mutation accumulation in duplicated IR regions. Using a Pi threshold of >0.015, we identified four highly variable regions within the LSC region: *rpoB~trnC-GCA*, *petN~psbM*, *trnV-UAC~trnM-CAU*, and *trnP-UGG~psaJ*. These regions represent candidate sites for future DNA barcoding development in *Prunus* species [47]. Beyond their taxonomic utility, these SSR loci and highly variable regions may also have practical value in breeding programs. In particular, they provide candidate chloroplast markers for maternal lineage verification, cultivar authentication, and germplasm management, especially in cases where pedigree records are incomplete or the maternal origin of breeding materials is uncertain. However, their application in routine breeding practice will require further validation using broader germplasm collections and segregating populations.

### 4.4. Phylogenetic Relationships and Taxonomic Implications

Although the taxonomic status of most species within the genus *Prunus* has been relatively well-established, the origin and classification of the apricot–plum hybrid have remained long-standing subjects of debate [48,49,50]. Based on phylogenetic analysis of the chloroplast genome, this study clearly divided *Prunus* species into four major clades, with high support values at key nodes. The phylogenetic tree revealed that ‘Flavor King’, ‘Flavor Supreme’, ‘Flavor Queen’, and *P. ussuriensis* form one cluster, while ‘Flavorosa’, ‘Dinosaur Egg’, ‘Flavorich’, and *P. salicina* cluster together. None of the six pluot cultivars formed closely related branches with *P. armeniaca* or *P. sibirica*, suggesting that their maternal lineages are distantly related to the subgenus *Armeniaca*.

Previous breeding and molecular studies also support the distinct plum-biased genetic background of pluots. Guerrero et al. [51] noted that pluots were primarily developed by backcrossing plumcot hybrids with *P. salicina*, resulting in a theoretical genetic composition of approximately 75% *P. salicina* genome and 25% *P. armeniaca*. SSR marker results revealed that the detected allelic variation in the pluot cultivars ‘Flavorich’ and ‘Flavorosa’ predominantly originated from plum (*P. salicina*), with fewer shared or apricot-specific components; in population clustering, pluots (‘Flavorich’ and ‘Flavorosa’) did not form an independent branch but were interspersed with plum cultivars, exhibiting significantly smaller genetic distances to plum than to apricot [3]. These findings strongly align with the documented breeding history involving multiple rounds of plum backcrossing. Regarding functional trait-associated loci, Halász et al. [52] identified the S genotype of pluot cultivars represented by ‘Flavor Grenade’ and ‘Flavor King’ as SbSc through PCR amplification, S-RNase intron length detection, and sequence analysis. This places them within the second incompatibility group (Group II) of *Prunus*, aligning with some major cultivated *P. salicina* cultivars (e.g., ‘Black Amber’, ‘October Sun’). This indicates that apricot–plums retain typical *Prunus* genetic characteristics at key self-incompatibility loci, suggesting that interspecific hybridization has not disrupted the core conservation of the *Prunus* gametophyte self-incompatibility system. Notably, the chloroplast genome primarily reflects maternal genetic history. Therefore, the conclusions of this study should be interpreted as indicating that the aforementioned commercial pluot cultivars are maternally closer to either *P. ussuriensis* or *P. salicina*. Their phylogenetic relationships and component proportions at the nuclear genome level require further validation using whole-genome nuclear data. The repeated sentence at the end of this paragraph was removed.

Although the close maternal affinity of pluots to *P. salicina* and *P. ussuriensis* is not unexpected given their known breeding history, the present chloroplast genomic evidence provides more than a simple confirmation of previous hybridization records. Commercial pluot cultivars often have complex backcross histories, and their pedigrees may be incomplete, ambiguous, or difficult to verify using morphology alone. Because chloroplast genomes are maternally inherited and largely non-recombining, they provide a stable molecular framework for assigning maternal lineages in such complex breeding materials. This information can complement conventional pedigree records and help distinguish whether similar fruit phenotypes arose from comparable or different maternal backgrounds. The value of plastome phylogenomics for resolving maternal ancestry has also been demonstrated in other complex horticultural crops. For example, a recent chloroplast phylogenomic study of cultivated chrysanthemum, *Chrysanthemum × morifolium*, successfully identified its maternal ancestry and traced its relationship to wild progenitor lineages, despite the complex hybrid origin of cultivated chrysanthemums [53]. This parallel case supports the broader applicability of complete chloroplast genomes for tracing maternal lineages in horticultural crops with complicated domestication or breeding histories. Together with the present results, it indicates that plastome-scale data can provide an independent and robust framework for maternal-lineage assignment, particularly when nuclear backgrounds have been reshuffled by repeated hybridization, backcrossing, or selection.

From a breeding perspective, the plastome-based grouping of the six cultivars into *P. ussuriensis*-associated and *P. salicina*-associated maternal lineages may help refine the use of maternal parents in future cross design. For example, when breeding objectives include cold tolerance, environmental adaptability, or stable performance in arid and semi-arid regions, maternal lines related to *P. ussuriensis* may warrant further evaluation because of the ecological adaptation of this species. Conversely, *P. salicina*-related maternal lineages may be useful for maintaining desirable fruit-quality traits associated with cultivated plum backgrounds. Therefore, the main applied value of the chloroplast data lies in converting a broadly expected breeding assumption into a testable maternal-lineage framework that can support parental selection, pedigree verification, and germplasm management.

In addition, this study revealed several inconsistencies with traditional taxonomic treatments: *P. sibirica*, traditionally placed in subg. *Armeniaca*, clustered within subg. *Prunus* in our phylogenetic tree; *P. tianshanica* and *Prunus tomentosa*, usually assigned to subg. *Cerasus*, were nested within the subg. *Armeniaca* clade. Cui et al. [10] also suggested a close relationship between *P. tomentosa* and subg. *Armeniaca* and recommended placing it within subg. *Armeniaca*. Regarding the taxonomic status of *P. padus*, our results support its placement in subg. *Cerasus* within *Prunus*, rather than recognizing it as a separate genus Padus, consistent with the conclusions of Bortiri et al. [54].

### 4.5. Time of Differentiation and Evolutionary History

Divergence time estimates indicate that the radiation of *Prunus* s.l. commenced during the Late Cretaceous, with a divergence time of approximately 63.83 Mya. This finding aligns with the hypothesis that paleoclimate changes drove the radiation of plant lineages [55]. The phylogenetic origin time (stem age) of subgenus *Cerasus* was estimated at 54.82 Mya in the early Eocene, closely matching the origin time (approximately 54.97 Mya) proposed by Yi et al. [56], and supports the establishment of early phylogenetic structure during the Eocene. Molecular clock analysis based on chloroplast whole genomes indicates that the two major lineages, dominated by *P. ussuriensis* and *P. salicina*, diverged around 34.28 Mya (95% HPD: 30.09–39.00 Mya), corresponding to the Late Oligocene. Because this node was directly constrained by a TimeTree-derived secondary calibration in the revised analysis, this estimate should be interpreted as a calibrated plastid lineage age rather than an independent species divergence time. This timing is significantly later than the early radiation event within *Prunus* (e.g., 44.06 Mya as estimated in this study) but precedes the large-scale climatic fluctuations of the Quaternary glacial period (Pleistocene, 2.6 Mya) [57], suggesting that the foundation for the divergence of the maternal ancestors of *P. ussuriensis* and *P. salicina* may have been established prior to the Quaternary. From a geological-climatic perspective, this period aligns with the global cooling trend and intensification of the East Asian monsoon during the Late Miocene [58].

In the phylogenetic topology, the six pluot cultivars studied (‘Flavor King’, ‘Flavor Supreme’, ‘Flavor Queen’, ‘Flavorosa’, ‘Dinosaur Egg’, ‘Flavorich’) are all tightly nested within the core clade formed by *P. salicina* and *P. ussuriensis*, without forming a closely related cluster with *P. armeniaca*. This pattern aligns with the maternal inheritance characteristic of chloroplast genomes: as artificially hybridized cultivars between plum and apricot, pluots can integrate parental components in their nuclear genomes, while chloroplast genomes are primarily inherited from the maternal lineage. Despite marked differences in commercial types and fruit phenotypes among the six cultivars, their genetic differentiation at the chloroplast genomic level is low, with short branch lengths in the phylogenetic tree and divergence times far less than 1 Mya. This result aligns with findings from Kim et al. [59] on the Korean pluot cultivar ‘Harmony’, suggesting that the commercial pluot cultivars studied here likely originate from a relatively closely related *Prunus* maternal lineage. In summary, this study uses chloroplast molecular clock analysis to reveal the close maternal affinity between commercial pluot cultivars, *P. ussuriensis*, and *P. salicina*, providing temporal evidence for the historical differentiation of their maternal lineages.

## 5. Conclusions

This study completed the whole-genome sequencing and assembly of chloroplast genomes of six commercial pluot cultivars. Comparative genomics and phylogenetic analyses were conducted in combination with other representative *Prunus* species. The results indicated that the overall structure of *Prunus* chloroplast genomes was highly conserved, with genome lengths varying only slightly (157,865–158,131 bp), though species-specific variations were observed at the IR boundaries. Based on sequence variation analysis, four highly variable regions (*rpoB~trnC-GCA*, *petN~psbM*, *trnV-UAC~trnM-CAU*, and *trnP-UGG~psaJ*) and 370 SSRs were identified as candidate chloroplast molecular markers for species identification, cultivar authentication, maternal lineage tracing, and genetic diversity analysis within *Prunus*. Phylogenetic analysis clearly delineated four subgeneric clades within *Prunus*, with stable topology and high support values. Among the six pluot cultivars, ‘Flavor King’, ‘Flavor Supreme’, and ‘Flavor Queen’ clustered with *P. ussuriensis*, while ‘Flavorosa’, ‘Dinosaur Egg’, and ‘Flavorich’ clustered with *P. salicina*, indicating that their chloroplast maternal lineages are closer to plum lineages within *Prunus*. Our results provide chloroplast-based insights into maternal lineage relationships in commercial pluot cultivars, but their application in breeding strategies should be further evaluated through broader sampling and integration with nuclear genomic data.

## Figures and Tables

**Table 1:** Summary of the cp genomes features of five *Prunus* spp.

Items		‘Flavor Supreme’	*P. ussuriensis*	*P. salicina*	*P. sibirica*	*P. armeniaca*
Total Size (bp)		157,924	157,918	157,916	158,138	157,865
LSC (bp)		86,187	86,187	86,123	86,358	86,217
SSC (bp)		19,031	19,025	19,029	19,120	18,988
IR (bp)		26,353	26,353	26,382	26,373	26,373
GC (%)	Total (%)	36.72%	36.72%	36.75%	36.70%	36.74%
	LSC (%)	34.51%	34.51%	34.58%	34.52%	34.55%
	SSC (%)	30.37%	30.37%	30.39%	30.30%	30.50%
	IR (%)	42.62%	42.62%	42.59%	42.57%	42.58%
Gene number		131	132	131	132	131
PCG		86	85	85	86	86
tRNA		37	39	38	38	37
rRNA		8	8	8	8	8

Large single-copy, LSC; small single-copy, SSC; inverted repeat sequence, IR; guanine-cytosine content, GC; protein-coding gene, PCG. Among them, pluot cultivar ‘Flavor Supreme’ specifically refers to the cultivar ‘Weidi’.

**Table 2:** Genes present in the chloroplast genomes of the five *Prunus* spp.

Category	Gene Group	Gene Name
Photosynthesis	Subunits of photosystem I	*psaA*, *psaB*, *psaC*, *psaI*, *psaJ*
	Subunits of photosystem II	*psbA*, *psbB*, *psbC*, *psbD*, *psbE*, *psbF*, *psbH*, *psbI*, *psbJ*, *psbK*, *psbL*, *psbM*, *psbN*, *psbT*, *psbZ*
	Subunits of NADH dehydrogenase	ndhA *, ndhB *(2), *ndhC*, *ndhD*, *ndhE*, *ndhF*, *ndhG*, *ndhH*, *ndhI*, *ndhJ*, *ndhK*
	Subunits of cytochrome b/f complex	*petA*, petB *, petD *, *petG*, *petL*, *petN*
	Subunits of ATP synthase	*atpA*, *atpB*, *atpE*, atpF *, *atpH*, *atpI*
	Large subunit of rubisco	*rbcL*
	Subunits photochlorophyllide reductase	-
Self-replication	Proteins of large ribosomal subunit	*rpl14*, rpl16 *, *rpl2(2)*, *rpl20*, *rpl22*, *rpl23(2)*, *rpl32*, *rpl33*, *rpl36*
	Proteins of small ribosomal subunit	*rps11*, rps12 **(2), *rps14*, *rps15*, rps16 *, *rps18*, *rps19(2)*, *rps2*, *rps3*, *rps4*, *rps7(2)*, *rps8*, *#rps19* ^3^
	Subunits of RNA polymerase	*rpoA*, *rpoB*, rpoC1 *, *rpoC2*
	Ribosomal RNAs	*rrn16S(2)*, *rrn23S(2)*, *rrn4.5S(2)*, *rrn5S(2)*
	Transfer RNAs	trnA-UGC *(2), *trnC-GCA*, *trnD-GUC*, *trnE-UUC*, *trnF-GAA*, *trnG-GCC*, trnG-GCC *, *trnH-GUG*, *trnI-CAU(2)*, trnI-GAU *(2), trnK-UUU *, *(trnS-GGA)* *trnL-CAA(2)*, trnL-UAA *, *trnL-UAG*, *trnM-CAU*, *trnN-GUU(2)*, *trnP-UGG*, *trnQ-UUG*, *trnR-ACG(2)*, *trnR-UCU*, *trnS-GCU(2)*, *trnS-UGA*, *trnT-GGU*, *trnT-UGU*, *trnV-GAC(2)*, trnV-UAC *, *trnW-CCA*, *trnY-GUA*, *trnfM-CAU*, *trnR(2)* ^2^
Other genes	Maturase	*matK*
	Protease	clpP **
	Envelope membrane protein	*cemA*
	Acetyl-CoA carboxylase	*accD*
	C-type cytochrome synthesis gene	*ccsA*
	Translation initiation factor	*infA* ^1^
	other	-
Genes of unknown function	Conserved hypothetical chloroplast ORF	*ycf1(2)*, *ycf2(2)*, ycf3 **, *ycf4*, *#ycf1* ^4^

Gene *: Contains one intron; Gene **: Contains two introns; #Gene: Pseudogene; Gene (2): Copy number of multicopy genes. Gene ^1^ and Gene ^2^: Specific genes of *P. ussuriensis*. Gene ^3^: Pseudogene in *P. salicina*. Gene ^4^: Pseudogene in *P. sibirica* and *P. armeniaca*.

## Data Availability

No datasets were generated or analyzed during the current study.

## Supplementary Materials

The following supporting information can be downloaded at: https://www.mdpi.com/article/10.3390/genes17060607/s1.

## Abbreviations

The following abbreviations are used in this manuscript:

LSC	Large single-copy
SSC	Small single-copy
IR	Inverted repeat sequence
GC	Guanine-cytosine content
PCG	Protein-coding gene
SSRs	Simple sequence repeats
BI	Bayesian Inference
ML	Maximum Likelihood
HPD	highest posterior density
Mya	million years ago
