# Supplementary material for: Comparative Chloroplast Genomics Reveals the Maternal Origin and Evolutionary Relationships of Commercial Pluot Cultivars Within Prunus
Source: Genes (Basel). 2026 May 27;17(6):607. doi: 10.3390/genes17060607 (PMC13299551; doi:10.3390/genes17060607)
Supplement: Supplementary file 1 [file genes-17-00607-s001.zip › genes-4315943-supplementary.docx]

| Number | Species | Accession |
| --- | --- | --- |
| 1 | *Prunus armeniaca* | KY101151.1 |
| 2 | *Prunus sibirica* | KY101154.1 |
| 3 | *Prunus mume* | MH700953.1 |
| 4 | *Prunus zhengheensis* | NC_062793.1 |
| 5 | *Prunus mandshurica* | MW599985.1 |
| 6 | *Prunus padus* | NC_026982.1 |
| 7 | *Prunus humilis* | NC_035880.1 |
| 8 | *Prunus salicina* | NC_047442.1 |
| 9 | *Prunus domestica* | NC_050959.1 |
| 10 | *Prunus cerasifera* | OM214582.1 |
| 11 | *Prunus divaricata* | PV138221 |
| 12 | *Prunus tianshanica* | PV138221 |
| 13 | *Prunus ussuriensis* | MK905688.1 |
| 14 | *Prunus dulcis* | MT019559.1 |
| 15 | *Prunus persica* | MW042696.1 |
| 16 | *Prunus davidiana* | NC_039735.1 |
| 17 | *Prunus pseudocerasus* | NC_030599.1 |
| 18 | *Prunus tomentosa* | NC_036394.1 |
| 19 | *Prunus cerasus* | MT576894.1 |
| 20 | *Prunus avium* | MK622380.1 |
| 21 | *Malus prattii* | MH929090.1 |
| 22 | *Crataegus pinnatifida* | NC_057086.1 |

**Table S1** Species information and accession numbers of the chloroplast genomes used in the phylogenetic analysis

| Items |  | 'Flavor Queen' | 'Flavorosa' | 'Dinosaur Egg' | 'Flavor King' | 'Flavorich' |
| --- | --- | --- | --- | --- | --- | --- |
| Total Size (bp) |  | 157 924 | 157 916 | 157 916 | 157 924 | 157 916 |
| LSC (bp) |  | 86 187 | 86 123 | 86 123 | 86 187 | 86 123 |
| SSC (bp) |  | 19 031 | 19 029 | 19 029 | 19 031 | 19 029 |
| IR (bp) |  | 26 353 | 26 382 | 26 382 | 26 535 | 26 382 |
| GC (%) | Total (%) | 36.72% | 36.75% | 36.75% | 36.72% | 36.75% |
|  | LSC (%) | 34.51% | 34.58% | 34.58% | 34.51% | 34.58% |
|  | SSC (%) | 30.37% | 30.39% | 30.39% | 30.37% | 30.39% |
|  | IR (%) | 42.62% | 42.59% | 42.59% | 42.62% | 42.59% |
| Gene number |  | 131 | 131 | 131 | 131 | 131 |
| PCG |  | 86 | 86 | 86 | 86 | 86 |
| tRNA |  | 37 | 37 | 37 | 37 | 37 |
| rRNA |  | 8 | 8 | 8 | 8 | 8 |

Table S2 Summary of the cp genomes features of five pluots

A

B

C

D

E

**Figure S1** Chloroplast genome maps of five pluots. A, Chloroplast genome map of pluot cultivar 'Flavor Queen'; B, Chloroplast genome map of pluot cultivar 'Flavorosa'; C, Chloroplast genome map of pluot cultivar 'Dinosaur Egg'; D, Chloroplast genome map of pluot cultivar 'Flavor King'; E, Chloroplast genome map of pluot cultivar 'Flavorich'.

**Figure S2** DNA agarose gel electrophoresis image. DNA Marker: 15000 bp. Lanes 1–6 represent samples of ‘Flavorich’, ‘Flavor Queen’, ‘Flavorosa’, ‘Flavor King’, ‘Dinosaur Egg’ and ‘Flavor Supreme’, respectively.

A

B

**Figure S3** Construction of a phylogenetic tree of the *Prunus* species. based on the maximum likelihood (A) and Bayesian (B) methods. A, The values of the nodes in the graph are bootstrap support values; B, the node values in the figure are the posterior probabilities values.
